# Degradation efficiencies of 2,4,6-TCP by Fe^0^-based advanced oxidation processes (AOPs) with common peroxides

**DOI:** 10.1371/journal.pone.0257415

**Published:** 2021-09-22

**Authors:** Luoyan Ai, Tiancheng Ren, Qin Yan, Mengdan Wan, Yajuan Peng, Xiaoyun Xu, Xinxin Liu

**Affiliations:** 1 School of Human Habitation and Environment, Nanchang Institute of Science and Technology, Jiangxi, Nanchang, China; 2 Jiangxi Academy of Transportation Science, Jiangxi, Nanchang, China; Indian Institute of Technology Bombay, INDIA

## Abstract

Degradation of 2,4,6-trichlorophenol (2,4,6-TCP) by zero-valent iron (ZVI) activating three common peroxides (peroxymonosulfate (PMS), hydrogen peroxide (H_2_O_2_), and peroxydisulfate (PS)) was investigated. The effects of ZVI dosage, peroxides concentration, initial pH, and Cl^-^ concentration were examined. The 2,4,6-TCP degradation efficiencies by Fe^0^/peroxides (PMS, H_2_O_2_, PS) were compared. Results showed that the order for degradation efficiency was H_2_O_2_≥PMS>PS. The degradation efficiency of 2,4,6-TCP in ZVI/peroxides systems were optimal at c(Ox) = 1 mmol•L^-1^; c(Fe^0^) = 0.1 g/L; initial pH = 3.2. Additionally, pH had a vital effect on 2,4,6-TCP degradation. At pH<3.2, ferrous play a vital role in all reaction, and accelerate the reaction rate rapidly. The existence of NaCl showed different results in the four systems. Chloride had little effect on 2,4,6-TCP degradation when chloride concentration at 5 mM, whereas the presence of 300 mM chloride significantly accelerated the degradation of 2,4,6-TCP from 72.7% to 95.2% in ZVI-PMS system. Notably, the other three systems showed opposite results. In contrast, the AOX (Absorbable Organic Halogen) values were highest in ZVI-PMS-Cl^-^ system, due to the formation of lots of refractory chlorinated phenols as identified by GC-MS. These findings are good for choosing the most suitable technology for chlorophenol wastewater treatment.

## 1 Introduction

Chlorophenols are widely used in different kinds of areas, such as wood preservers, pesticides, herbicides, biocides, and dyes [1, 2]. due to the numerous sources, chlorophenols have been found in groundwater and wastewater [3]. Chlorophenols are persistent, hardly biodegradable and easily accumulate in the environment. These substances have been reported to have adverse effects on the nervous system and have been connected to many health disorders. 2,4,6-TCP has been designated as a priority pollutant, and listed in the Drinking Water Contaminant Candidate List (CCL) [4, 5].

Several treatment technologies have been proposed to degrade chlorophenols, including activated carbon adsorption, biological treatment, incineration adsorption, and air stripping [2, 4, 6]. However, these techniques have various limitations and defects. Like biological treatment for chlorinated phenols degradation have been proposed inefficient since chlorinated phenols are easily inhibit microorganisms [7, 8].

The limitations of traditional technology to degrade chlorophenols have led to efforts to explore alternative methods such as advance oxidation processes (AOPs) [9]. One cost-efficient approach for chlorophenols degradation is reductive dechlorination using zero-valent metals (ZVMs). In several ZVMs, iron has been extensively applied to chlorinated hydrocarbon dechlorination because of its abundant reserves and environmental-friendly [10–13]. To the best of our knowledge, many researchers have worked on the oxidation of organic by Fe^0^-based advance AOPs. Hou et al. [14] illustrated the effectiveness of using ZVI-based Fenton process for treating rhodamine. Ghanbari et al. [15] also investigated the decolorization of textile wastewater by ZVI activated peroxymonosulfate, the research also compared with zero valent copper (ZVC), the result showed ZVI compared ZVC was more effective in terms of COD and color removals. Oh et al. [16] reported spent caustic degradation using Fenton and persulfate oxidation with ZVI, spent caustic was mineralized when iron powder added into hydrogen peroxide or persulfate solution. In addition, these researches have showed the oxidation caused by free radicals which were generated by ZVI activating hydrogen peroxide (H_2_O_2_, E^0^(H_2_O_2_/H_2_O) = 1.77 V vs NHE (Normal Hydrogen Electrode)), peroxydisulfate (PS, S_2_O_8_^2-^, E^0^(S_2_O_8_^2-^/SO_4_^2-^) = 2.01 V vs NHE), and peroxymonosulfate (PMS, HSO_5_^-^, E^0^(HSO_5_^-^/HSO_4_^-^) = 1.85 V vs NHE). Current studies have proven the effectiveness of ZVI as an activator in peroxygen oxidation [17–20]. Whereas, comparative research on 2,4,6-TCP by ZVI-based peroxygen activation has not been reported. ZVI as a reducing agent can lead to the dechlorination of 2,4,6-TCP; furthermore, the existence of peroxygens can further decompose 2,4,6-TCP to other chlorinated compounds. In such complex systems, we cannot yet determine the degradation mechanism of 2,4,6-TCP. Therefore, in this study, the ZVI loading, solution pH, peroxygen concentration, sodium chloride dose, AOX value, and 2,4,6-TCP degradation products are evaluated to explore the different effectiveness of ZVI-based peroxygen systems. AOX is a measurement for halogenated compounds and is an important parameter for the characterization of industrial wastewaters. The value of AOX showed the eco-toxicity of water-contaminated. Therefore, the best technology for contaminant elimination with ZVI-based peroxygen systems can be chosen from an environmentally friendly point of view.

## 2 Experimental

### 2.1 Materials

Iron powder, hydrogen peroxide (30%, v/v), sodium chloride (NaCl), sodium hydroxide (NaOH), and sulfuric acid (H_2_SO_4_, 98%) were purchased from Sinopharm. 2,4,6-TCP was from Acros Organics. Oxone^®^ (2KHSO_5_•KHSO_4_•K_2_SO_4_) was obtained from Sigma-Aldrich. Potassium persulfate (K_2_S_2_O_8_) was bought from Alfa Aesar. Methanol (HPLC grade) was obtained from CNW Technologies GmbH. All chemical reagents were used as received. All reaction solutions were prepared in deionized water.

### 2.2 Experimental procedures

**Fig 1:**
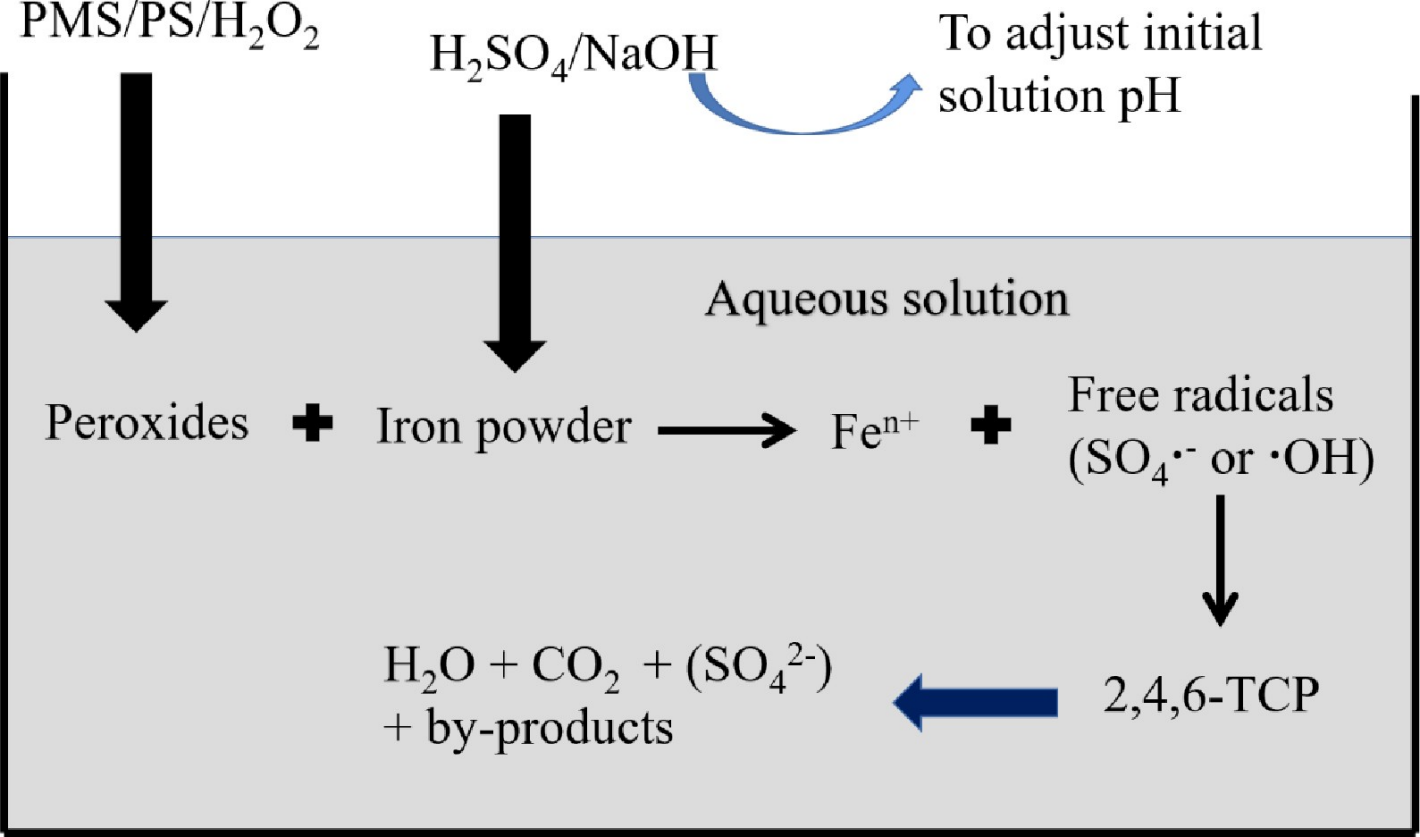
The scheme of peroxides activation by ZVI.

All experiments were conducted at room temperature in a 100 mL glass vessel using a total volume of 50 mL under constant stirring with a magnetic stirring apparatus. The scheme of peroxides activation by Fe^0^ catalysts is schematically illustrated in Fig 1. For the experiment, the initial concentration of 2,4,6-TCP was 0.2 mmol•L^-1^, and 0.1 mol•L^-1^ sulfuric acid or 0.1 mol•L^-1^ sodium hydroxide was used to adjust the initial solution pH values. At selected time intervals (0, 5, 10, 15, 20, and 30 min), a 1-mL sample was collected from each reaction solution and immediately quenched with methanol. The quenched sample was passed through a 0.22 μm membrane filter before HPLC analysis. For the measurement of AOX and products, samples were quenched by sodium sulfate and sodium nitrite, respectively, at a ratio of 1:1. All experiments were prepared in duplicate to ensure reproducibility and to estimate experimental errors.

### 2.3 Analysis

The concentration of 2,4,6-TCP was measured by HPLC (Prominence LA-20A) equipped with a 5 μm J&KChemica^®^ C_18_ column (4.6×250 mm) and was detected by its corresponding peaks at 290 nm. Mixtures of methanol (85%) and water (15%) were used as the mobile phase at a flow rate of 0.8 mL/min for 2,4,6-TCP.

For the identification of 2,4,6-TCP and its degradation products, liquid samples after extraction, concentration and silylation were analyzed with gas chromatography-mass spectrometry. AOX detection was carried out by analytical instruments (AOX, Multi X 2500, Jena, Germany) after sample pretreatment.

A pseudo first-order kinetic model is used to describe the degradation kinetics in Fe^0^/peroxides systems. The kinetic expression is represented as Eq 1, C_0_ is the 2,4,6-TCP initial concentration, C_t_ is the residual 2,4,6-TCP concentration at time *t* (min). k denotes the observed pseudo first-order rate constant (min^-1^). the constant *k* is calculated by the slope of a plot of ln(C_t_/C_0_) versus t and is summarized in Table 1.


$$C_{t}/C_{0}=e^{‐\mathrm{kt}}$$ (1)

**Table 1:** The calculated pseudo first-order rate constant of 2,4,6-TCP degradation in Fe^0^-peroxides systems.

		Fe		Fe/H_2_O_2_		Fe/PMS		Fe/PS	
		*k*	R^2^	*k*	R^2^	*k*	R^2^	*k*	R^2^
pH^a^	2	5.5*10^−3^	0.928	7.3*10^−2^	0.994	8.6*10^−2^	0.961	1.6*10^−2^	0.984
	3.2	3.4*10^−3^	0.944	5.8*10^−2^	0.958	5.3*10^−2^	0.975	2.4*10^−2^	0.916
	5.0	-	-	1.9*10^−3^	0.942	2.1*10^−2^	0.997	2.2*10^−2^	0.954
	7.0	-	-	-	-	5.2*10^−3^	0.874	1.4*10^−2^	0.982
iron dosage (g/L)^b^	0.02	2.2*10^−3^	0.823	2.4*10^−2^	0.984	4.9*10^−3^	0.921	1.1*10^−2^	0.990
	0.06	2.3*10^−3^	0.860	6.1*10^−2^	0.951	1.8*10^−2^	0.995	1.4*10^−2^	0.936
	0.1	4.0*10^−3^	0.968	6.5*10^−2^	0.820	6.1*10^−2^	0.913	1.5*10^−2^	0.898
	0.2	1.8*10^−3^	0.702	5.0*10^−2^	0.649	8.7*10^−2^	0.977	1.2*10^−2^	0.838
Peroxygens concentration (mmol•L^-1^)^d^	0.2	-	-	1.4*10^−2^	0.896	1.0*10^−2^	0.995	4.4*10^−3^	0.861
	0.6	-	-	3.8*10^−2^	0.771	2.8*10^−2^	0.972	1.0*10^−2^	0.830
	1.0	-	-	5.9*10^−2^	0.926	5.1*10^−2^	0.934	1.5*10^−2^	0.904
	2.0	-	-	0.14	0.874	3.4*10^−2^	0.956	2.3*10^−2^	0.878
Cl^-^concentration (mmol•L^-1^)^e^	0	2.1*10^−3^	0.962	5.1*10^−2^	0.988	4.8*10^−2^	0.943	1.4*10^−2^	0.943
	5	1.0*10^−3^	0.630	5.2*10^−2^	0.988	3.0*10^−2^	0.999	1.3*10^−2^	0.871
	100	-	-	3.4*10^−2^	0.980	5.3*10^−2^	0.944	-	-
	300	-	-	2.7*10^−3^	0.999	0.15	0.801	-	-

^a^Conditions: c(2,4,6-TCP) = 0.2 mmol•L^-1^; c(Ox) = 1 mmol•L^-1^; c(Fe^0^) = 0.1 g/L.

^b^Conditions: c(2,4,6-TCP) = 0.2 mmol•L-1; c(Ox) = 1 mmol•L-1; pH = 3.2.

^d^Conditions: c(2,4,6-TCP) = 0.2 mmol•L-1; c(Fe0) = 0.1 g/L; pH = 3.2. ^e^Conditions: c(2,4,6-TCP) = 0.2 mmol•L-1; c(Ox) = 1 mmol•L-1; c(Fe^0^) = 0.1 g/L; pH = 3.2.

## 3 Results and discussion

### 3.1 Effect of Fe^0^ dose on 2,4,6-TCP oxidation

**Fig 2:**
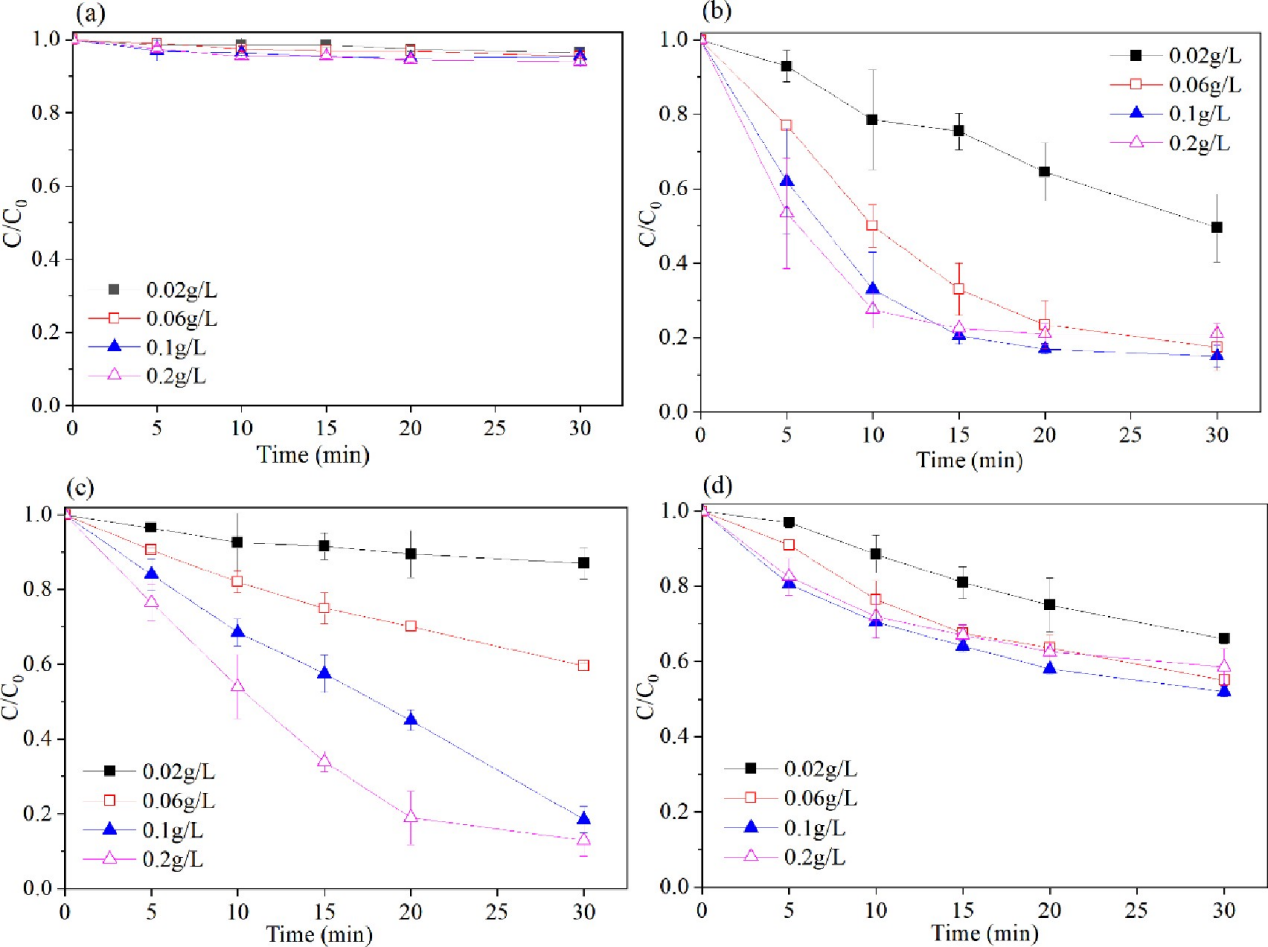
Effect of iron loading on the degradation of 2,4,6-TCP in four systems. (a) Fe^0^; (b) Fe^0^/H_2_O_2_; (c) Fe^0^/PMS; (d) Fe^0^/PS. Conditions: c(2,4,6-TCP) = 0.2 mmol•L^-1^; c(Ox) = 1 mmol•L^-1^; pH = 3.2±0.05.

Experiments were used to confirm the influence of the ZVI amount on the degradation efficiency of 2,4,6-TCP by changing the ZVI loading while keeping the peroxides concentration constant at 1 mM and the 2,4,6-TCP concentration at 0.2 mM. The doses of ZVI were 0.02, 0.06, 0.1, and 0.2 g/L. As shown in (Fig 2), normally, the degradation ratio increased with an increasing mass of added ZVI. When 0.02 to 0.2 g/L ZVI was used, 2,4,6-TCP degradation proceeded in a gradual and sustained manner in the Fe^0^/H_2_O_2_, Fe^0^/PS and Fe^0^/PMS systems. The degradation ratio increased with an increasing iron dose in the Fe^0^/peroxide systems. Fe^0^ can generate dissolved Fe^2+^, which can also activate peroxides in acidic conditions (Eqs 2–8). When 0.2 g/L Fe^0^ was used, the 2,4,6-TCP oxidation rates were all over 80% in the Fe^0^/H_2_O_2_ and Fe^0^/PMS systems, and a 37% 2,4,6-TCP degradation ratio was found in the Fe^0^/PS system. However, without the presence of peroxides, ZVI could hardly decompose 2,4,6-TCP.


$$Fe^{0}+2H^{+}\to Fe^{2+}+H_{2}$$ (2)

$$Fe^{2+}+H_{2}O_{2}\to Fe^{3+}+•OH+OH^{‐}$$ (3)

$$2Fe^{3+}+Fe^{0}\to 3Fe^{2+}$$ (4)

$$Fe^{0}+S_{2}O_{8}{}^{2‐}\to Fe^{2+}+2SO_{4}{}^{.‐}+2SO_{4}{}^{2‐}$$ (5)

$$Fe^{2+}+S_{2}O_{8}{}^{2‐}\to Fe^{3+}+SO_{4}{}^{.‐}+SO_{4}{}^{2‐}$$ (6)

$$Fe^{2+}+HSO_{5}{}^{‐}\to SO_{4}{}^{.‐}+OH^{‐}+Fe^{3+}$$ (7)

$$Fe^{0}+HSO_{5}{}^{‐}\to Fe^{3+}+SO_{4}{}^{2‐}+•OH$$ (8)

### 3.2 Effect of the initial concentrations of peroxides on 2,4,6-TCP degradation

**Fig 3:**
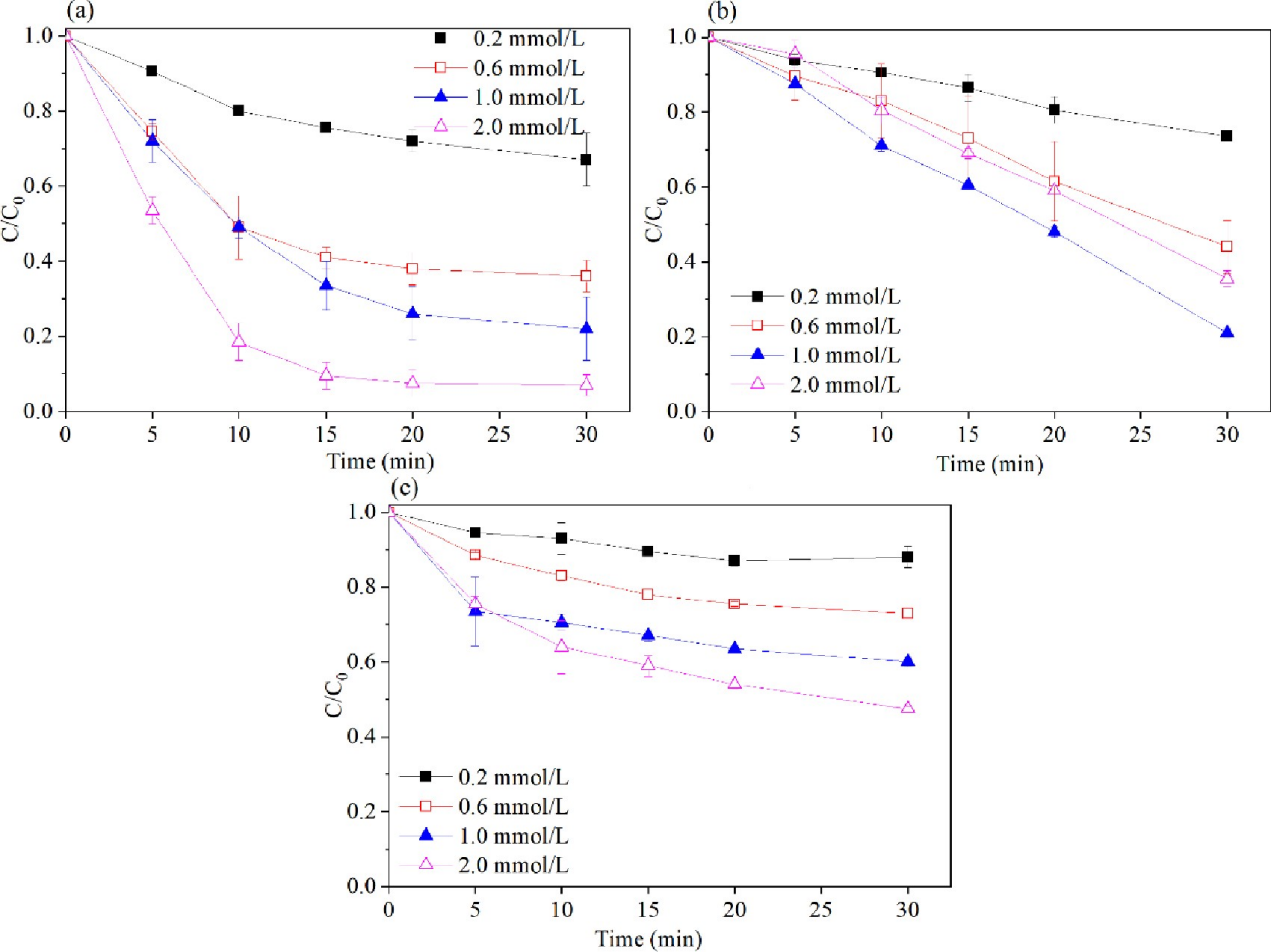
Effect of peroxides concentration on the degradation of 2,4,6-TCP in four systems. (a) Fe^0^/H_2_O_2_; (b) Fe^0^/PMS; (c) Fe^0^/PS. Conditions: c(2,4,6-TCP) = 0.2 mmol•L^-1^; c(Fe^0^) = 0.1 g/L; pH = 3.2±0.05.

The amount of peroxides used is a vital factor with respect to 2,4,6-TCP degradation. In this study, peroxide concentrations of 0.2, 0.6, 1.0 and 2.0 mmol•L^-1^ were investigated in four systems, and the results are shown in (Fig 3). The results indicate that the degradation rate of 2,4,6-TCP remarkably increased with an increasing peroxide concentration. This result suggests that Fe^0^ could activate peroxides to generate living free radicals, such as •OH and SO_4_^.-^ (Eqs 2–8) [21–24]. 2,4,6-TCP was almost completely degraded when the H_2_O_2_ concentration was increased to 2.0 mmol•L^-1^. However, in the Fe^0^/PMS system, when the PMS dose increased from 1.0 mmol•L^-1^ to 2.0 mmol•L^-1^, the 2,4,6-TCP degradation rate decreased from 80% to 64%. This result was because excess PMS could react with living radicals •OH and SO_4_^.-^ (Eqs 9 and 10) [25, 26].


$$HSO_{5}{}^{‐}+•OH\to SO_{5}{}^{.‐}+OH^{‐}$$ (9)

$$HSO_{5}{}^{‐}+SO_{4}{}^{.‐}\to SO_{4}{}^{2‐}+H^{+}$$ (10)

### 3.3 Effect of the initial pH on 2,4,6-TCP degradation

**Fig 4:**
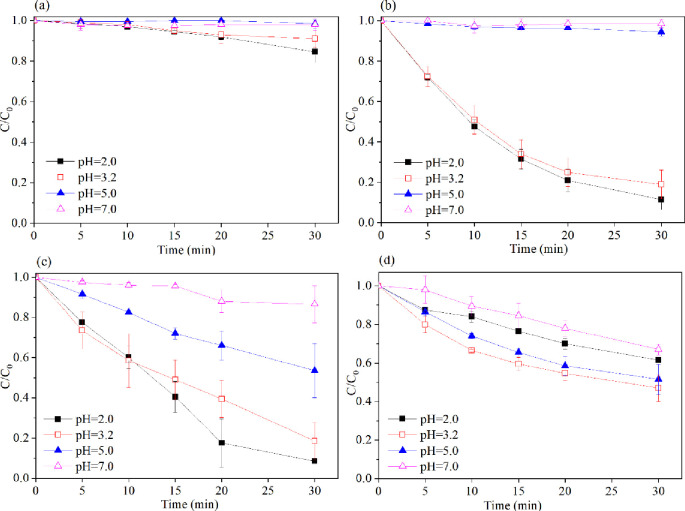
Effect of initial pH on the degradation of 2,4,6-TCP in four systems. (a) Fe^0^; (b) Fe^0^/H_2_O_2_; (c) Fe^0^/PMS; (d) Fe^0^/PS. Conditions: c(2,4,6-TCP) = 0.2 mmol•L^-1^; c(Ox) = 1 mmol•L^-1^; c(Fe^0^) = 0.1 g/L.

The time evolution of the 2,4,6-TCP concentration ratios (C/C_0_) with different initial pH values is shown in (Fig 4). This figure shows that the initial solution pH is a critical parameter in ZVI systems. The experiments were carried out at pH values of 2, 3.2, 5 and 7. Initially, the pH of the solution was 3.2 without any pH adjustment in the ZVI/PMS system. Under the other conditions, the pH was adjusted using H_2_SO_4_ or NaOH. Zero-valent iron can be converted to ferrous ions at low pH values. Thus, in Fig 4(A), when the pH was adjusted from 2 to 7, the degradation efficiency of 2,4,6-TCP decreased from 16% to 0. Fig 4(B) shows that the degradation rate of 2,4,6-TCP increased with a decreasing initial solution pH, and the best degradation of 89% was achieved at pH 2 within 30 min of reaction. This result is in agreement with a previous study for a Fenton-like system: the ferrous-ion concentration increases under acidic conditions and reacts with hydrogen peroxide to generate OH radicals (Eqs 1 and 2) [23]. At higher pH values, the 2,4,6-TCP degradation rate decreased rapidly when pH>3.2. This result was not only ascribed to the decomposition of H_2_O_2_ but also to the deactivation of ZVI with the formation of Fe^3+^-hydroxo complexes in the reaction [23, 27–29]. As shown in Fig 4(C), oxidation process reached highest degradation efficiency (91.2% in 30 min reaction time) at pH = 2. Ghanbari et al. [15] indicated that ferrous ions played a vital role at pH<3.2 in ZVI/PMS system. At pH>3.2, the formation of Fe^3+^-hydroxide complexes, which are highly stable, made it difficult to catalyze PMS to generate sulfate radicals [30]. We can see from Fig 3(D) that the effective condition for 2,4,6-TCP degradation was at pH 3.2, with approximately 53% of 2,4,6-TCP being removed within 30 min. However, 2,4,6-TCP degradation was poor under extremely acidic conditions. Only 38% of 2,4,6-TCP was degraded at pH 2 within 30 min. Though the conversion of Fe^0^ to Fe^2+^ by H^+^ (Eq 2) was rapid, acid catalysis (Eqs 11 and 12) would promote the formation of sulfate radicals; thus, the many sulfate radicals in the ZVI/PS system accelerated the self-scavenging of SO_4_^.-^ (Eq 13) and S_2_O_8_^2-^ (Eq 14). Therefore, 2,4,6-TCP degradation was critically slowed [31, 32].


$$S_{2}O_{8}{}^{2‐}+H^{+}\to HS_{2}O_{8}{}^{‐}$$ (11)

$$HS_{2}O_{8}{}^{‐}\to H^{+}+SO_{4}{}^{.‐}+SO_{4}{}^{2‐}$$ (12)

$$SO_{4}{}^{.‐}+SO_{4}{}^{.‐}\to S_{2}O_{8}{}^{2‐}$$ (13)

$$SO_{4}{}^{.‐}+S2O_{8}{}^{2‐}\to SO_{4}{}^{2‐}+S_{2}O_{8}{}^{‐}$$ (14)

### 3.4 Effect of Cl^-^ concentration on 2,4,6-TCP degradation

**Fig 5:**
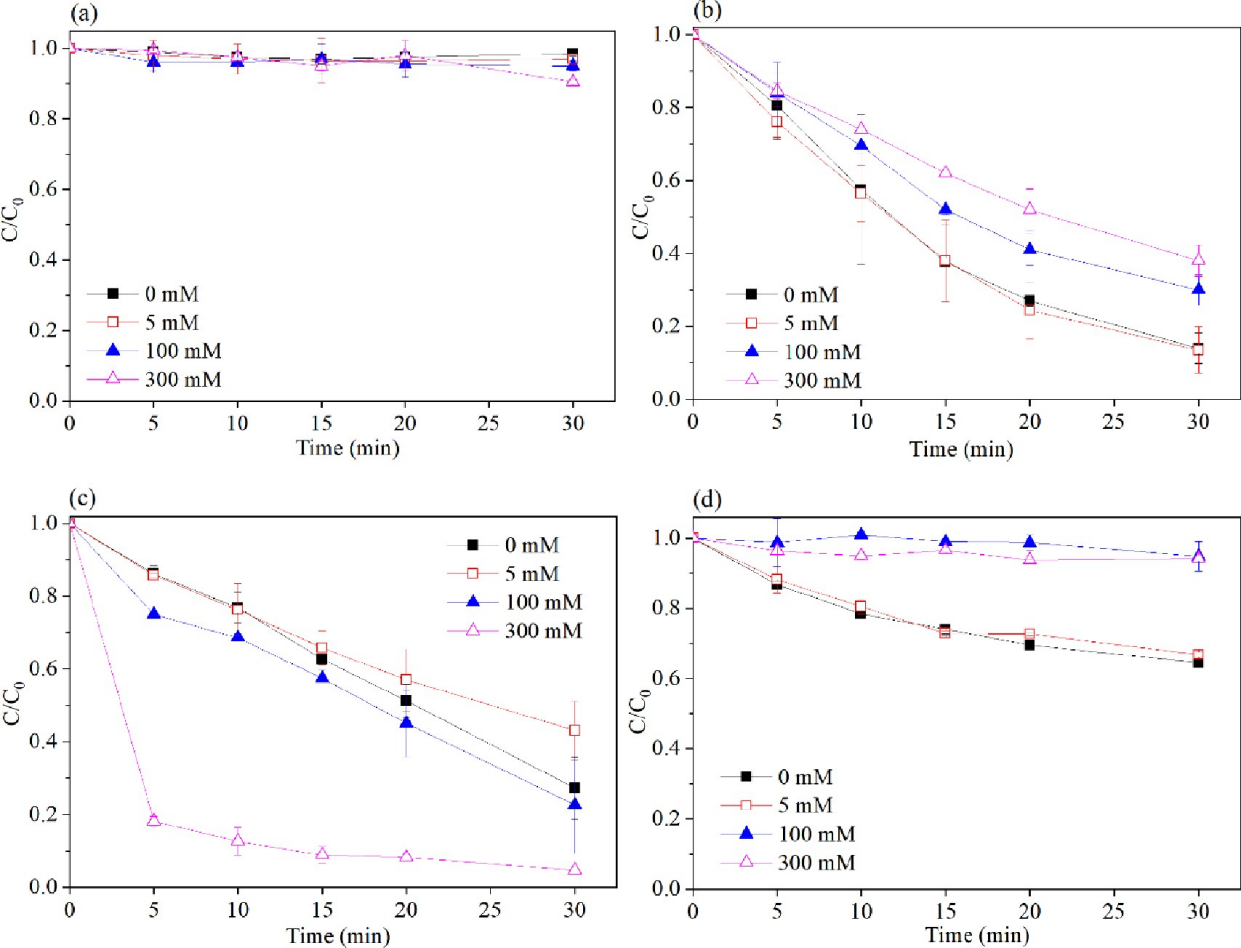
Effect of chloride ions concentration on the degradation of 2,4,6-TCP in four systems. (a) Fe^0^; (b) Fe^0^/H_2_O_2_; (c) Fe^0^/PMS; (d) Fe^0^/PS. Conditions: c(2,4,6-TCP) = 0.2 mmol•L^-1^; c(Ox) = 1 mmol•L^-1^; c(Fe^0^) = 0.1 g/L; pH = 3.2±0.05.

Dyeing wastewater contains many chloride ions with high concentrations of biorefractory organic compounds [33, 34]. The effect of Cl^-^ on 2,4,6-TCP degradation by ZVI/peroxide oxidation was investigated. The experiments were conducted in the chloride-ion concentration range of 0–300 mM. The results are shown in (Fig 5). Fig 5(A) shows that chloride ions have a negligible effect on the 2,4,6-TCP degradation efficiency. We found remarkably significant similarities among the ZVI/H_2_O_2_ and ZVI/PS systems. When the chloride-ion concentration was 5 mM, 2,4,6-TCP degradation was not significantly influenced. However, as the concentration of chloride ions was increased to 300 mM, the inhibition of 2,4,6-TCP degradation was observed. This result was because chloride ions scavenged the hydrogen and sulfate radicals [34]. Similar studies on the addition of chloride ions have been reported on ozonation [35], UV-H_2_O_2_ [36], and UV-TiO_2_ processes [37]. In the ZVI/PMS system, when the chloride-ion concentration was below 5 mM, the slight inhibitory effect of 2,4,6-TCP degradation was investigated, and it was thermodynamically feasible for sulfate radicals (2.5–3.1 V) to oxidize chloride ions into less reactive radicals, viz., 2Cl^-^/Cl_2_ (1.36 V) and Cl^-^/HOCl (1.48 V) (Eqs 15–19) [25, 33]. When the chloride-ion concentration was increased to 100 and 300 mM, an acceleration in the degradation of 2,4,6-TCP was found. This result was ascribed to PMS oxidizing Cl^-^ into Cl_2_ and HOCl (Eqs 20 and, 21), which showed high oxidation ability when coexisting [38–40].


$$Cl^{‐}+SO_{4}{}^{.‐}\to SO_{4}{}^{2‐}+Cl•$$ (15)

$$Cl•+Cl^{‐}\to Cl_{2}{}^{.‐}$$ (16)

$$Cl_{2}{}^{.‐}+Cl_{2}{}^{.‐}\to Cl_{2}+2Cl^{‐}$$ (17)

$$Cl_{2}+H_{2}O\to HOCl+H^{+}+Cl^{‐}$$ (18)

$$HOCl\to H^{+}+OCl^{‐}$$ (19)

$$HSO_{5}{}^{‐}+Cl^{‐}\to SO_{4}{}^{2‐}+HOCl$$ (20)

$$HSO_{5}{}^{‐}+2Cl^{‐}+H^{+}\to SO_{4}{}^{2‐}+Cl_{2}+H_{2}O$$ (21)

### 3.5 GC-MS analysis

**Fig 6:**
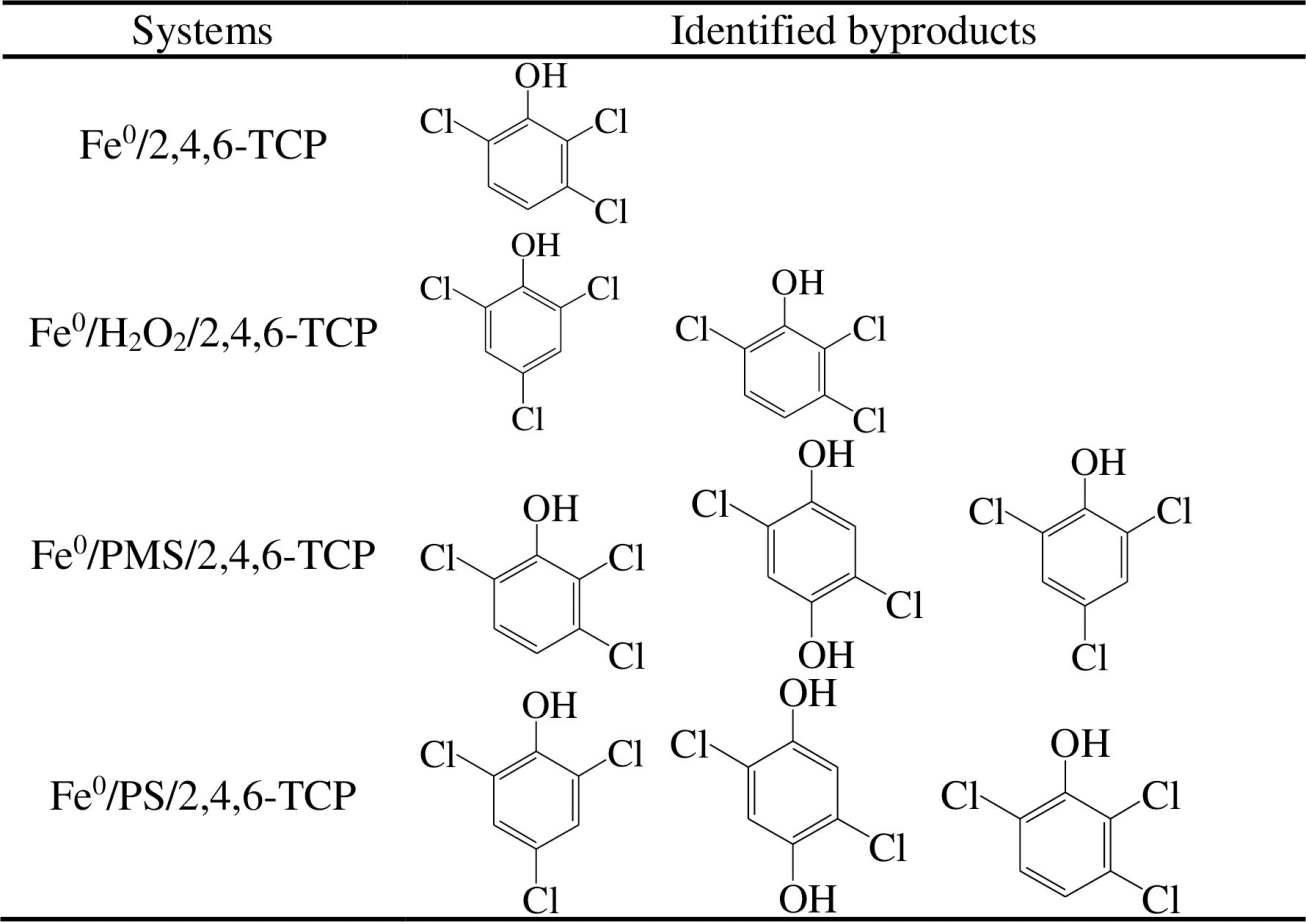
The transformation products of 2,4,6-TCP degradation in four systems (without Cl^-^).

**Fig 7:**
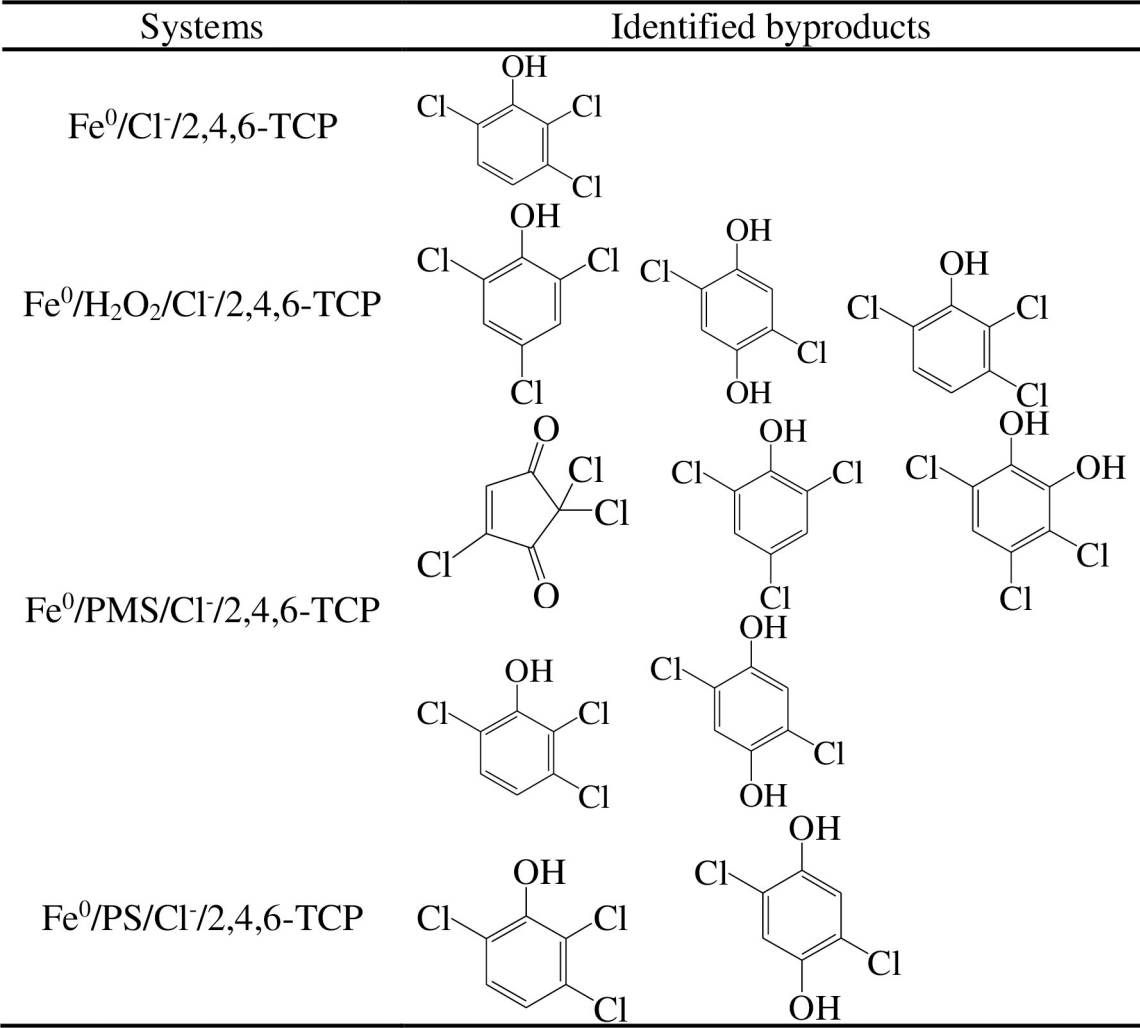
The transformation products of 2,4,6-TCP degradation in four systems (with Cl^-^).

We learned the kinetics of the four systems, and the intermediates during the degradation of 2,4,6-TCP by ZVI/peroxide were assessed and presented in this section. Without the presence of chloride, the intermediates of the four systems were similar (Fig 6). In the ZVI/PMS and ZVI/PS systems, we detected the same intermediates, viz., 2,5-dichloro-benzene-1,4-diol and 2,3,6-trichloro-phenol, and the unreacted contaminant. In the ZVI/H_2_O_2_ and ZVI systems, we only found 2,3,6-trichlorophenol. Therefore, in actual wastewater treatment, we prefer to use the ZVI/H_2_O_2_ or ZVI/PMS system to decompose targeted contaminants. In high salinity wastewater, due to the high concentration of chloride ions, there are different results. The concentration of chloride ions in the reaction was 300 mM. From Fig 7, we know that in the ZVI/PS, ZVI/H_2_O_2_, and ZVI systems, the existence of chloride had negligible influence on the intermediates of the 2,4,6-TCP degradation process. However, in the ZVI/PMS system, chloride ions were added to the solution, and the byproduct species in 2,4,6-TCP degradation increased rapidly. The intermediates of 2,4,6-TCP degradation by ZVI/PMS were 2,3,6-trichloro-phenol, 2,5-dichloro-benzene-1,4-diol, 2,2,4-trichloro-cyclopent-4-ene-1,3-dione, 2,4,6-trichloro-phenol, 3,4,6-trichloro-benzene-1,2-diol, and 2,5-dichloro-benzene-1,4-diol.

### 3.6 AOX formation

**Fig 8:**
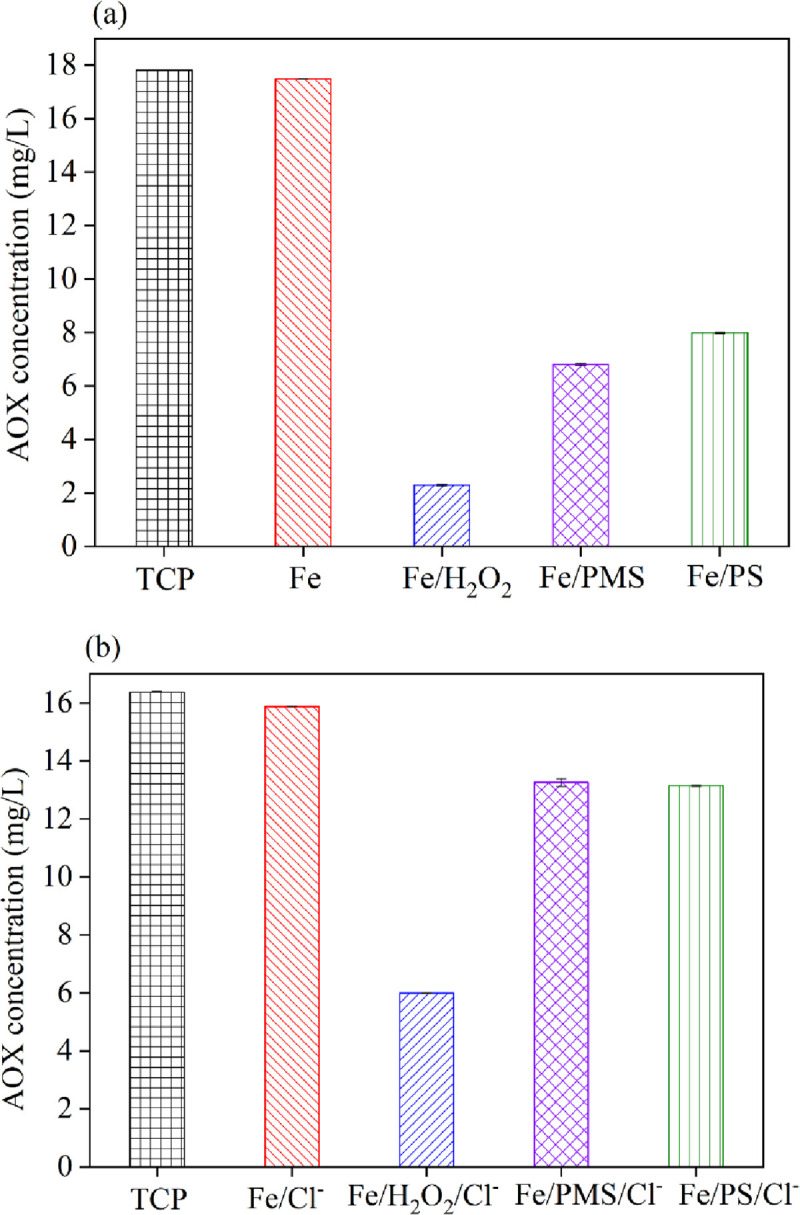
AOX concentration of 2,4,6-TCP degradation in four systems within 30 min. (a) without Cl^-^; (b) with Cl^-^. Conditions: c(2,4,6-TCP) = 0.2 mmol•L^-1^; c(Ox) = 1 mmol•L^-1^; c(Fe^0^) = 0.1 g/L; pH = 3.2±0.05; c(NaCl) = 300 m.

Adsorbable organic halogens (AOX) are a significant parameter for industrial wastewater and are used to measure halogenated compounds. Chloride ions tend to react with free radicals to generate chloride radicals, causing the formation of chloroform and halogenated derivatives [33, 41]. To confirm the effect of chloride ions on AOX changes, experiments were carried out at chloride-ion concentrations of 0 mM and 300 mM. (Fig 8) shows the results of AOX measurements in the four systems. The 2,4,6-TCP AOX value was detected by the initial 2,4,6-TCP solution [42]. Fig 8(A) shows that without the existence of chloride, the AOX concentration was Fe^0^>Fe^0^/PS>Fe^0^/PMS>Fe^0^/H_2_O_2_, according to the GC-MS results. However, Fig 8(B) shows that when the concentration of chloride ions was 300 mM, the AOX concentration increased in all four systems, especially in the Fe^0^/PMS system. This result indicated that the existence of chloride could result in the formation of halogenated derivatives, which matched with the GC-MS conclusions. When used to treat high salinity wastewater, the ZVI/PMS system is not a suitable method. A Fenton or Fenton-like system is a more suitable choice.

## 4 Conclusions

In this study, commercially available iron powder was used to activated three common peroxides (H_2_O_2_, PMS and PS) for the degradation of a typical chlorophenols (i.e., 2,4,6-TCP). Experimental results indicated ZVI could activate peroxides efficiently at acidic pH to degrade chlorophenols. The highest 2,4,6-TCP degradation was achieved in ZVI/H_2_O_2_ system, meanwhile, caused the lowest AOX value. However, ZVI/PMS and ZVI/PS systems showed higher 2,4,6-TCP degradation efficiency than ZVI/H_2_O_2_ system at pH >3.2. Increasing iron dose and peroxides concentration favored a rapid degradation of 2,4,6-TCP in ZVI/H_2_O_2_ and ZVI/PS systems. The presence of high chloride concentration (300 mM) promoted 2,4,6-TCP degradation rapidly in ZVI/PMS system, whereas 2,4,6-TCP degradation was inhibited in ZVI/H_2_O_2_ and ZVI/PS systems. Nevertheless, more refractory by-products, like 2,3,6-trichlorophenol, 3,6-dichlorohydroquinone, 3,4,6-trichlorocatechol and other chlorinated compound were found by GC-MS, which also reflected in AOX results. In conclusion, ZVI/H_2_O_2_ system was more flexible and beneficial in acidic and high saline wastewater treatment, while ZVI/PS and ZVI/PMS technologies are more suitable for low salinity wastewater treatment.
